# Association Between Serum 25(OH)D Concentrations and Glucose-Lipid Metabolism in Women With Gestational Diabetes Mellitus: Analysis of A NHANES 2007–2018

**DOI:** 10.33549/physiolres.935791

**Published:** 2026-06-01

**Authors:** Fang ZHANG, Ling WANG, Zhi-Yu DONG, Shu-Han LV, Xue-Ju ZHENG, Li-Min CHEN, Xue-Fei YUAN, Hui-Jing SHI

**Affiliations:** 1 The First College of Clinical Medicine, Guizhou University of Traditional Chinese Medicine, Guiyang Guizhou, China; 2 Department of Obstetrics, The First Affiliated Hospital of Guizhou, University of Traditional Chinese Medicine, Guiyang Guizhou, China; 3 East Hospital Affiliated to Tongji University, Shanghai, China; 4 Department of Traditional Chinese Medicine Gynecology, Taizhou Hospital of Traditional Chinese Medicine, Taizhou, Zhejiang, China; 5 Department of Gynecology, The First Affiliated Hospital of Guizhou, University of Traditional Chinese Medicine, Guiyang Guizhou, China; 6 Center for Reproductive Medicine of Changhai Hospital, Naval Medical University, Shanghai, China

**Keywords:** 25-hydroxyvitamin D, Dose-response relationship, Gestational diabetes mellitus, Insulin resistance, Lipid metabolism

## Abstract

Vitamin D is implicated in multiple metabolic processes, however, the dose-dependent relationship between serum 25-hydro-xyvitamin D [25(OH)D] concentrations and glucose-lipid metabolism remains unclear among women with a history of gestational diabetes mellitus (GDM). This study aimed to assess both linear and non-linear relationships between serum 25(OH)D levels and indicators of insulin resistance and lipid metabolism in this high-risk group. Data from 1,876 women with prior GDM were obtained from the 2007–2018 National Health and Nutrition Examination Survey (NHANES). Weighted linear regression models were applied to assess associations between serum 25(OH)D and metabolic markers, while restricted cubic spline models were used to examine non-linear relationships. Analyses were adjusted for age, ethnicity, body mass index (BMI), education, and lifestyle factors. Following multivariate adjustment, each 10 nmol/l increase in serum 25(OH)D was associated with a decrease in HOMA-IR by 0.017 units (95 % CI: −0.026, −0.007), an increase in HDL-C by 0.127 mg/dl (95 % CI: 0.102, 0.153), and a reduction in total cholesterol of 0.007 mmol/l (95 % CI: −0.009, −0.004). Restricted cubic spline analysis demonstrated a U-shaped relationship between 25(OH)D and LDL-C (p for non-linearity<0.001), with inflection point at ~50 nmol/l. The inverse association between 25(OH)D and HOMA-IR was significant in non-Hispanic white individuals (β=−0.020, p=0.008) but not in non-Hispanic Black individuals (β=0.009, p=0.584). The positive association between 25(OH)D and HDL-C was strongest in women with normal BMI (β=0.114). Among women with a history of GDM, serum 25(OH)D levels were inversely associated with insulin resistance and positively associated with HDL-C. A non-linear, U-shaped relationship was observed with LDL-C. These associations varied by race/ethnicity and BMI, the potential relevance of vitamin D status in in the long-term metabolic management of individuals following GDM.

## Introduction

Gestational Diabetes Mellitus (GDM), the most prevalent metabolic complication of pregnancy, is characterized by glucose intolerance with onset or first recognition during gestation. GDM is associated with adverse short-term pregnancy outcomes and an elevated long-term risk of chronic metabolic diseases in both mothers and their offspring [1]. The global prevalence of GDM continues to rise, presenting a significant public health concern [2,3]. The underlying pathophysiology primarily involves a combination of progressive insulin resistance and inadequate compensatory insulin secretion by pancreatic β-cells, typically resulting in widespread disruptions in glucose and lipid metabolism [4].

Current clinical management strategies for GDM rely primarily on lifestyle interventions and insulin therapy; however, these approaches are limited by challenges such as poor adherence and inadequate long-term follow-up. Therefore, exploring innovative and effective therapeutic strategies and targets is key for the prevention and management of GDM. Vitamin D, a steroid hormone with multiple metabolic regulatory functions, has attracted increasing attention for its potential role in glucose and lipid metabolism [5]. Mechanistic studies have demonstrated that vitamin D modulates insulin gene expression and secretion through activation of the vitamin D receptor (VDR) and enhances insulin sensitivity in peripheral tissues [6–8]. In addition, hormonally active form of vitamin D, 1,25-dihydro-xyvitamin D_3_ (1,25(OH)_2_D_3_), regulates lipogenesis and lipolysis *via* both genomic and non-genomic pathways, thereby contributing to lipid homeostasis [9].

Prior studies have indicated a significant association between vitamin D deficiency during pregnancy and an increased risk of GDM development [10]. However, most available evidence has concentrated on risk prediction. Limited evidence is available regarding the extent to which vitamin D levels influences the severity of glucose-lipid metabolic disturbances among women with established GDM. Evidence from large-scale epidemiological datasets is particularly lacking. To address this gap, this study used data from the National Health and Nutrition Examination Survey (NHANES) to examine the association between serum 25(OH)D concentrations and key glucose-lipid metabolic indicators in women with a history of GDM. It was hypothesized that lower 25(OH)D levels would be significantly associated with an adverse metabolic profile. The findings provide epidemiological evidence regarding the role of vitamin D in the pathophysiology of GDM and may inform future therapeutic strategies targeting long-term cardiometabolic risk in this population.

## Participants and Methods

### Ethics approval and consent to participate

The NHANES protocols covering 2007–2018 were reviewed and approved by the National Center for Health Statistics Institutional Review Board (Protocols #2021-05, #2018-01, and #2011-17). Written informed consent was obtained from every participant. Because the data are de-identified and publicly released, the institutional review board of Guizhou Medical University exempted the present secondary analysis from additional ethical review and participant consent.

### Study design and data source

A secondary analysis was conducted using data obtained from the NHANES database. NHANES serves as a nationally representative surveillance program designed to assess health and nutritional parameters of the non-institutionalized civilian population in the United States. The survey is conducted by the National Center for Health Statistics (NCHS), a division of the Centers for Disease Control and Prevention. Detailed descriptions of the sampling methodology and data collection procedures have been previously documented [11]. All study protocols were approved by the NCHS Ethics Review Board, and written informed consent was obtained from all participants.

**Fig. 1:**
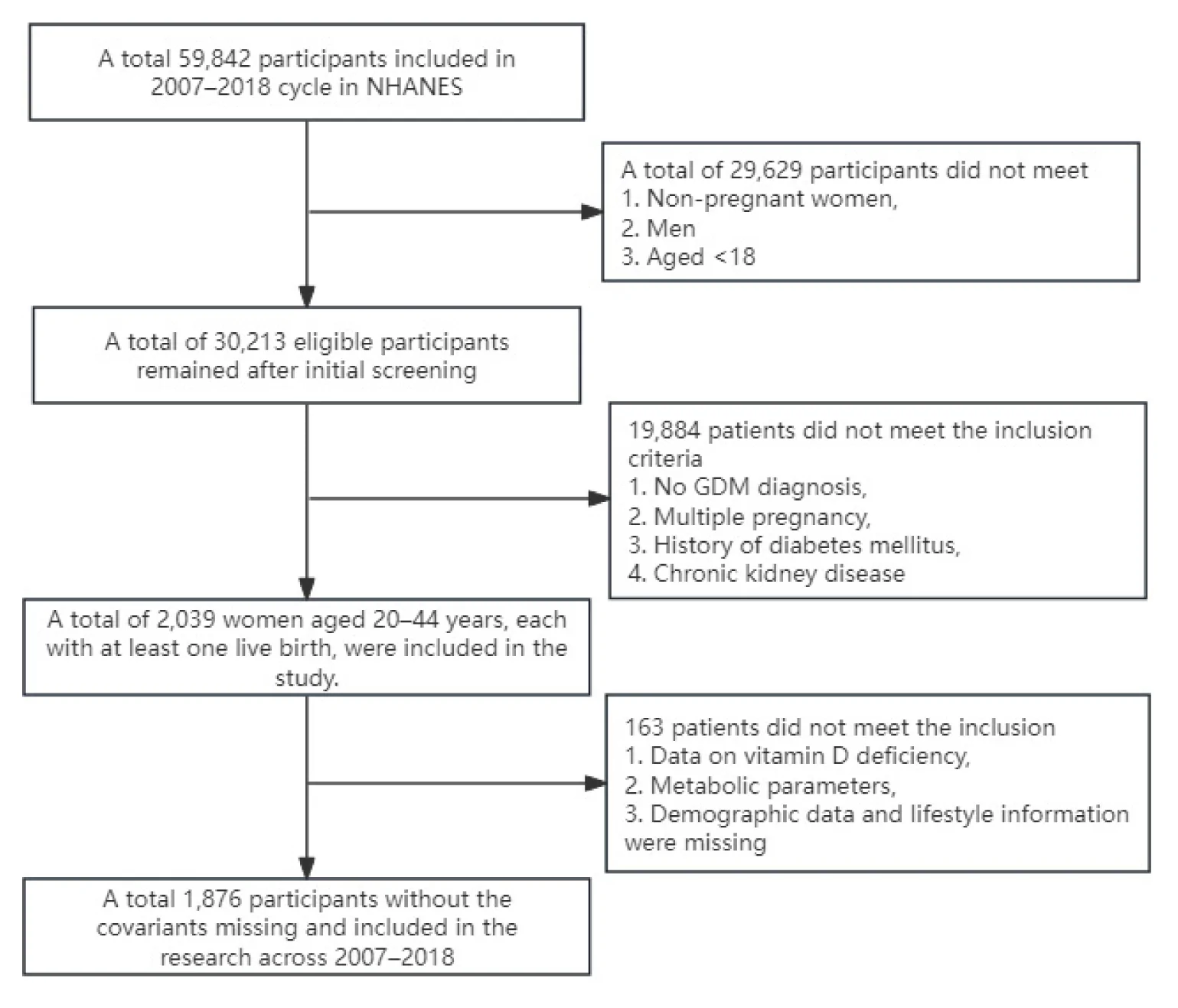
Flowchart of participant selection in National Health and Nutrition Examination Survey (NHANES) 2007–2018.

Data from six consecutive NHANES cycles (2007–2018) were included. The study population consisted of women with a history of GDM, identified through responses to questionnaire item DIQ175: “Have you ever been told by a doctor or other health professional that you had gestational diabetes?” Participants were excluded if they reported a diagnosis of diabetes prior to pregnancy, had a history of multiple gestation pregnancies, or chronic kidney disease. After application of these exclusion criteria, 1,876 eligible participants were included in the final analysis (Fig. 1).

### Measurement and definition of variables

During the 2007–2018 NHANES cycles included in this analysis, serum 25-hydroxyvitamin D [25(OH)D] concentrations were measured using a standardized liquid chromatography-tandem mass spectrometry (LC-MS/MS) assay.

Primary outcome variables consisted of a panel of glucose and lipid metabolism biomarkers obtained from fasting blood samples. These included fasting plasma glucose (FPG), total cholesterol (TC), triglycerides (TG), high-density lipoprotein cholesterol (HDL-C), and low-density lipoprotein cholesterol (LDL-C). Insulin resistance was estimated using the Homeostasis Model Assessment of Insulin Resistance (HOMA-IR), calculated as: [fasting plasma glucose (mmol/l) × fasting insulin (μU/ml)]/22.5. All procedures related to the collection, processing, and analysis of biological specimens followed standardized and validated protocols.

Covariate data were obtained through structured household interviews and physical assessments. Demographic variables included age (analyzed as a continuous variable) and race/ethnicity, categorized as non-Hispanic Black, Mexican American, non-Hispanic White, and other racial/ethnic groups. Socioeconomic status was assessed through educational attainment (categorized as less than high school, high school graduate or equivalent, and college or higher) and household financial resources. The poverty-income ratio (PIR) was stratified into three levels: <1.3, 1.3–3.5, and >3.5.

Lifestyle-related variables included tobacco use (categorized as never, former, or current), alcohol consumption (based on self-reported frequency and quantity over the past 12 months), and physical activity (assessed by type, frequency, and duration). Clinical variables included body mass index (BMI) and reproductive history. BMI was calculated from height and weight measurements obtained at Mobile Examination Centers, expressed in kg/m^2^. Parity data were collected *via* self-report questionnaires.

### Statistical analysis

All analyses accounted for the complex, multistage probability sampling design of the NHANES dataset. Appropriate sampling weights, stratification variables, and primary sampling unit identifiers were applied to ensure nationally representative estimates. Weighted multivariable linear regression models were utilized to assess associations between serum 25-hydro-xyvitamin D [25(OH)D] concentrations and glucose-lipid metabolism biomarkers. Three hierarchical models were constructed: Model 1 was unadjusted; Model 2 adjusted for demographic covariates, including age, race/ethnicity, educational attainment, and household income; and Model 3 was further adjusted for lifestyle-related factors (smoking status, alcohol consumption, physical activity), BMI, and parity.

To explore potential non-linear dose-response relationships between serum 25(OH)D concentrations and metabolic outcomes, restricted cubic spline (RCS) regression with three knots was applied within the fully adjusted model. Non-linearity was assessed using likelihood ratio tests, comparing models containing only linear terms to those that included both linear and spline terms.

Effect modification was assessed through stratified analyses based on race/ethnicity and BMI category (<25 vs. ≥25 kg/m^2^). Interaction terms between serum 25(OH)D concentrations and each stratification variable were incorporated into the models, and the statistical significance of interaction effects was determined accordingly. All data processing and statistical analyses were conducted using Decision Chain software. A two-sided p-value of <0.05 was considered statistically significant.

## Results

### Baseline characteristics of the study population

A total of 1,876 women with a history of GDM were included in the analysis. The mean age of the cohort was 35.93±0.11 years and the mean BMI was 31.76±0.18 kg/m^2^ (Table 1). Participants were stratified into four groups based on serum 25(OH)D concentrations: severe deficiency (<25.0 nmol/l, n=312), moderate deficiency (25.0–49.9 nmol/l, n=643), insufficiency (50.0–74.9 nmol/l, n=576), and sufficiency (≥75.0 nmol/l, n=345).

Statistically significant differences were observed across groups in terms of race/ethnicity (p<0.001), BMI (p<0.001), poverty-income ratio (p<0.001), educational attainment (p=0.009), and physical activity participation (p=0.005). Among participants with serum 25(OH)D concentrations ≥75.0 nmol/l, 80.42 % identified as non-Hispanic White, compared with 32.08 % in the <25.0 nmol/l group. Mean BMI values for these respective groups were 29.52±0.37 kg/m^2^ and 34.78±0.44 kg/m^2^. Corresponding poverty-income ratios were 3.37±0.09 and 2.17±0.08, while the proportion reporting attainment of a college-level education or higher was 36.40 % and 14.70 %, respectively. Reported engagement in physical activity was 25.18 % in the ≥75.0 nmol/l group and 11.01 % in the <25.0 nmol/l group.

No statistically significant differences were observed across groups in terms of age distribution (p=0.756), alcohol consumption (p=0.234), smoking status (p=0.294), or reproductive history (p=0.374).

### Association between vitamin D levels and metabolic indicators

Table 2 presents least square means of glucose and lipid metabolic markers stratified by serum 25(OH)D concentration categories. After adjusting for age, race/ethnicity, BMI, educational attainment, alcohol consumption, smoking status, and reproductive history, significant associations were observed between serum 25(OH)D concentrations and Homeostasis Model Assessment of Insulin Resistance (HOMA-IR) (p=0.006), total cholesterol (p=0.006), and HDL-C (p<0.001).

Participants with serum 25(OH)D concentrations ≥75.0 nmol/l exhibited notably lower adjusted mean HOMA-IR values (4.93±0.20) and total cholesterol levels (5.88±0.06 mmol/l) compared to those with concentrations <25.0 nmol/l (HOMA-IR: 6.32±0.32; total cholesterol: 6.37±0.09 mmol/l). HDL-C concentrations increased progressively across higher 25(OH)D categories, ranging from 48.24±0.75 mg/dl in the severely deficient group (<25.0 nmol/l) to 57.26±0.89 mg/dl in the sufficient group (≥75.0 nmol/l).

No statistically significant differences were identified between groups with respect to fasting plasma glucose, LDL-C, or triglyceride concentrations.

Findings from the multivariable linear regression analysis (Table 3) were consistent with these patterns. In the fully adjusted model (Model 3), serum 25(OH)D concentrations of 50.0–74.9 nmol/l were associated with significantly lower HOMA-IR (β=−1.19; 95 % CI: −2.28, −0.10; p=0.028) and total cholesterol (β=−0.29; 95 % CI: −0.57, −0.01; p=0.041) compared with the baseline group (<25.0 nmol/l). Additionally, HDL-C concentrations were significantly higher in both the 50.0–74.9 nmol/l group (β=5.60; 95 % CI: 2.58, 8.62) and the ≥75.0 nmol/l group (β=7.47; 95 % CI: 4.01, 10.93) when compared to the reference group (p<0.001).

### Stratified analysis

Table 4 summarizes the results of stratified analyses examining the associations between serum 25(OH)D concentrations and metabolic indicators. When stratified by race/ethnicity, an inverse association between 25(OH)D concentrations and HOMA-IR was statistically significant among non-Hispanic White participants (β=−0.020; 95 % CI: −0.035, −0.005) but was not observed among non-Hispanic Black participants (β=0.009; 95 % CI: −0.023, 0.042).

Stratification by educational attainment indicated the strongest inverse association between serum 25(OH)D and HOMA-IR among participants with a high school diploma or General Educational Development certification (β=−0.042; 95 % CI: −0.072, −0.013). No significant association was observed in those reporting some college education or an associate degree (β=0.000; 95 % CI: −0.012, 0.012). The interaction between education level and the 25(OH)D-HOMA-IR association was statistically significant (interaction p=0.027).

The positive association between 25(OH)D concentrations and HDL-C was statistically significant across all racial/ethnic groups, with the strongest effect size observed among non-Hispanic White participants (β=0.159; 95 % CI: 0.115, 0.202).

In analyses stratified by BMI, an inverse association between 25(OH)D concentrations and total cholesterol was most significant among women with BMI ≥30 kg/m^2^ (β=−0.006; 95 % CI: −0.010, −0.002).

Stratification by smoking status indicated that the positive association between 25(OH)D and HDL-C was most pronounced among former smokers (β=0.164; 95 % CI: 0.130, 0.198). The interaction between smoking status and the 25(OH)D-HDL-C association was statistically significant (interaction p=0.011).

### Dose-response analysis

**Fig. 2:**
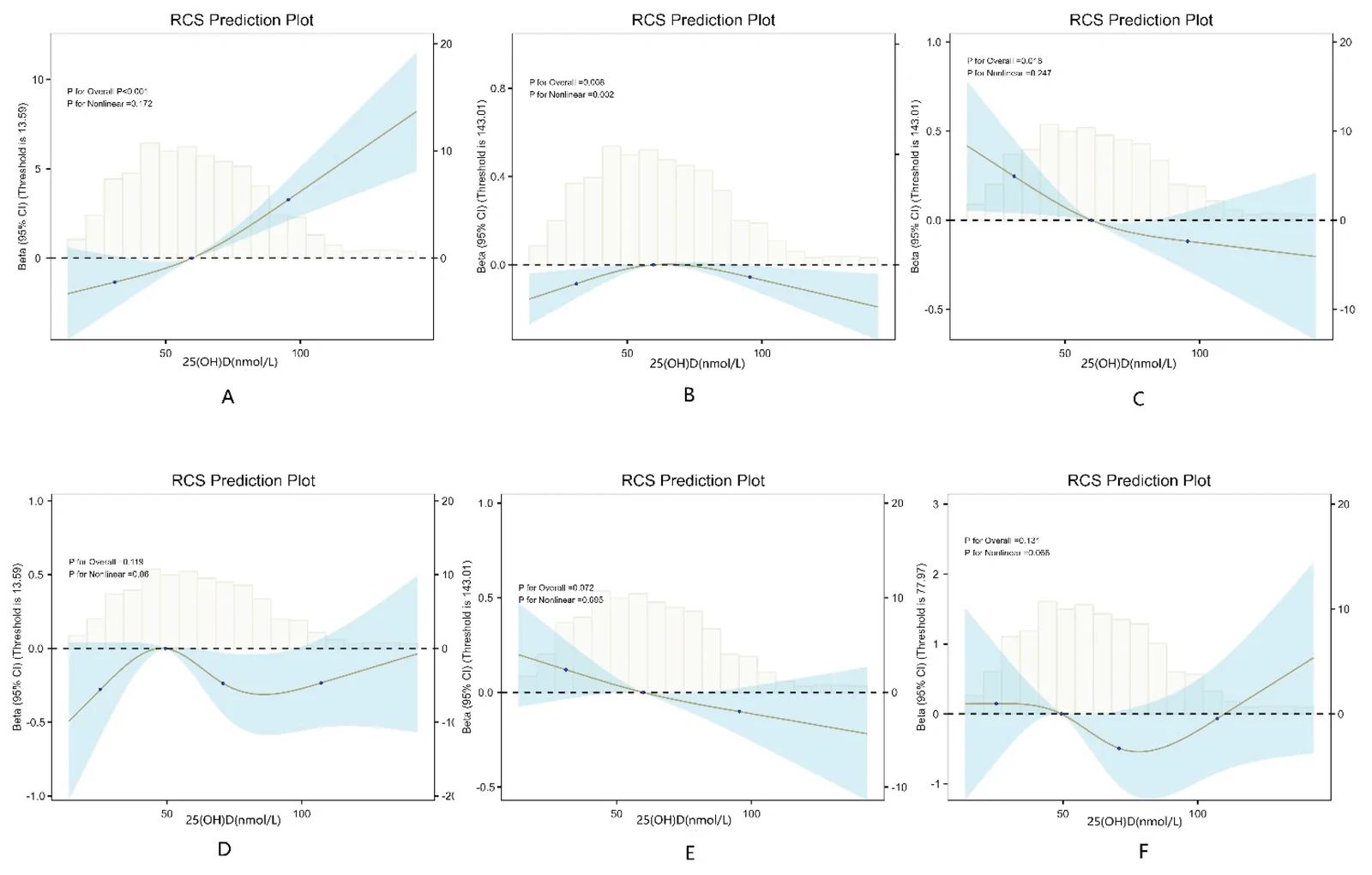
Dose-response relationships between serum 25(OH)D levels and metabolic indicators. Restricted cubic spline analyses showing associations between serum 25(OH)D levels and metabolic indicators. Solid lines represent point estimates and light blue areas represent 95 % confidence intervals. Histograms show the distribution of 25(OH)D. (**A**) 25(OH)D showed significant linear positive correlation with HDL-C. (**B**) 25(OH)D showed significant U-shaped relationship with LDL-C. (**C**) 25(OH)D showed negative correlation with triglycerides. (**D**) 25(OH)D showed complex nonlinear pattern with fasting glucose. (**E**) 25(OH)D showed negative correlation trend with HOMA-IR. (**F**) 25(OH)D showed U-shaped relationship trend with total cholesterol.

RCS models were applied to assess potential non-linear associations between serum 25(OH)D concentrations and various metabolic indicators, as depicted in Figure 2.

A significant positive, approximately linear positive association was observed between 25(OH)D concentrations and HDL-C levels (overall p<0.001; test for non-linearity, p=0.172; Fig. 2A). In contrast, the association between 25(OH)D and LDL-C followed a statistically significant U-shaped curve (overall p=0.008; non-linearity p=0.002; Fig. 2B).

A negative association was identified between 25(OH)D concentrations and triglyceride levels (overall p=0.018), although no significant non-linear pattern was found (non-linearity p=0.247; Fig. 2C). The relationship between 25(OH)D and fasting plasma glucose demonstrated a complex non-linear trend but did not reach statistical significance (overall p=0.119; non-linearity p=0.060; Fig. 2D). Similarly, the association between 25(OH)D and HOMA-IR exhibited a negative association but was not statistically significant (overall p=0.072; non-linearity p=0.895; Fig. 2E).

The association between 25(OH)D and total cholesterol exhibited a U-shaped pattern; however, neither the overall relationship (p=0.131) nor the non-linearity component (p=0.066) reached statistical significance (Fig. 2F).

## Discussion

The analysis of data from the 2007–2018 NHANES, including 1,876 women with a history of GDM, identified several associations between 25(OH)D concentrations and key metabolic parameters. The primary findings indicated an inverse association between 25(OH)D levels and insulin resistance, a positive association with HDL-C, and a U-shaped relationship with LDL-C. These associations varied across racial/ethnic groups and body composition categories.

The inverse association between 25(OH)D concentrations and HOMA-IR (β=−0.118; 95 % CI: −0.197, −0.039; p=0.003) is consistent with findings from prior studies, indicating that higher vitamin D levels may be linked to improved insulin sensitivity [12–13]. This relationship was further confirmed to be linear through RCS analysis (p for linearity=0.003; p for non-linearity=0.723), with no evidence of a threshold effect. In contrast, Jamka *et al*. reported differing results, which may be attributable to differences in study populations, sample sizes, or methodological approaches [14].

Stratified analysis revealed that the inverse association between 25(OH)D and HOMA-IR was more pronounced among non-Hispanic White participants (β=−0.165; p=0.008), while the association was not statistically significant among non-Hispanic Black participants (β=−0.074; p=0.213). This racial/ethnic variation aligns with findings reported by Yin *et al*., indicating that racial/ethnic background may serve as a modifying factor in the relationship between vitamin D status and insulin sensitivity [15]. This observed disparity may be driven by a combination of biological and environmental factors. A recent systematic review and meta-analysis by Abdul-Jabbar *et al*. [16] demonstrated that ethnicity significantly modifies the response to vitamin D supplementation. Their findings indicate that Black participants exhibit a blunted serum 25(OH)D response compared to White participants, even after adjusting for dosage and BMI. A primary mechanism for this heterogeneity likely lies in the genetic polymorphisms of Vitamin D Binding Protein (VDBP). As established by Powe *et al*. [17], Black Americans predominantly carry the GC-1F allele, whereas White Americans are more likely to carry the GC-1S and GC2 alleles. These distinct isoforms influence the bioavailability and transport of vitamin D, potentially limiting its metabolic efficacy in the Black population despite similar total serum levels. Additionally, Parva *et al*. [18] highlighted that higher melanin levels in darker skin reduce endogenous vitamin D synthesis, which, combined with dietary patterns, contributes to the severe deficiency observed in our Black cohort (Table 1).

Regarding lipid metabolism, a significant positive linear association was observed between 25(OH)D concentrations and HDL-C (β=5.341; 95 % CI: 2.674, 8.008; *p*<0.001), consistent with findings from Gunasegaran *et al*. who reported similar associations in a cohort of pregnant women in India [18]. In contrast, a U-shaped relationship was identified between 25(OH)D and LDL-C, with a statistically significant non-linear pattern (p for non-linearity=0.002) and an estimated inflection point near 50.0 nmol/l. Although this pattern has been infrequently described in previous studies, it may help explain inconsistencies in existing literature. It aligns with the hypothesis proposed by Mirhosseini *et al*., which proposes that the effect of vitamin D on lipoproteins may be subject to threshold dynamics [19]. The identified inflection point closely corresponds to the commonly used clinical threshold distinguishing vitamin D insufficiency from sufficiency (50 nmol/l), implying that the relationship between vitamin D status and lipid metabolism may be concentration dependent.

An inverse association was also observed between 25(OH)D concentrations and triglyceride levels (p overall=0.018; p for non-linearity=0.247). Meanwhile, associations with fasting plasma glucose and total cholesterol exhibited non-linear trends, though these did not reach statistical significance (p for non-linearity=0.060 and 0.066, respectively).

Stratified analysis by BMI indicated that the positive association between 25(OH)D concentrations and HDL-C was more pronounced among women with BMI≥25 kg/m^2^ (β=6.24; *p*<0.001) and attenuated among those with BMI<25 kg/m^2^ (β=3.17; p=0.083). These findings underscore the potential role of adiposity in modulating the absorption, distribution, and metabolic processing of vitamin D [20].

Several mechanistic studies offer biological explanations for the observed associations. Bland *et al*. reported that pancreatic islet cells express 1α-hydroxylase, an enzyme responsible for converting 25(OH)D] to its biologically active form, 1,25-dihydroxyvitamin D_3_ [1,25(OH)_2_D_3_] [19]. This peripheral enzymatic activity provides a mechanistic link between vitamin D status and pancreatic β-cell function. Experimental studies have further demonstrated that 1,25(OH)_2_D_3_ may reduce oxidative stress, inhibit β-cell apoptosis, and modulate calcium signaling pathways processes directly implicated in insulin secretion and sensitivity [21–25]. In parallel, the presence of VDR in adipocytes, along with the regulatory effects of vitamin D on parathyroid hormone and lipid-related gene expression, may help explain the observed associations between 25(OH)D concentrations and lipid parameters [26,27].

The U-shaped association identified between 25(OH)D and LDL-C may reflect a bidirectional regulatory role of vitamin D on lipid metabolism. At lower concentrations, vitamin D may reduce LDL-C levels by attenuating inflammation and oxidative stress. However, at concentrations above the identified inflection point (~50 nmol/l), compensatory mechanisms such as increased hepatic cholesterol synthesis may be activated, potentially leading to elevated LDL-C levels. This non-linear pattern indicates that vitamin D supplementation strategies in women with a history of GDM may require individualized approaches to avoid excessive dosing.

This study has several methodological strengths. The use of data from the nationally representative NHANES dataset enhances the generalizability of the findings. The application of RCS modeling enabled the identification of non-linear associations that would not have been detected using traditional linear regression approaches. Additionally, the analysis incorporated a comprehensive range of confounding variables and incorporated stratified evaluations, thereby improving the robustness and clinical relevance of the findings.

However, several limitations should be acknowledged. The cross-sectional study design limits the ability to infer causal relationships between serum 25-hydroxyvitamin D [25(OH)D] concentrations and metabolic outcomes. GDM status was determined based on self-reported data rather than clinical records, introducing the potential for misclassification. Additionally, data on the timing of pregnancy were not available, limiting adjustment for potential stage-specific metabolic variations. Additionally, 25(OH)D concentrations were assessed at a single-point, which may not accurately reflect long-term vitamin D status. Furthermore, although age and BMI were adjusted in our models to serve as proxies for menopausal status and adiposity, direct measurements of serum estrogen levels were not consistently available in the NHANES dataset. Given the known role of estrogen in regulating β-oxidation and lipid metabolism, residual confounding from unmeasured hormonal variations cannot be entirely ruled out. Finally, the potential influence of unmeasured or residual confounding, such as dietary vitamin D intake or genetic factors affecting vitamin D metabolism, cannot be entirely excluded.

## Conclusions

This study systematically examined the dose-response relationships between serum 25(OH)D concentrations and metabolic indicators in women with a history of GDM. The findings demonstrated an inverse association with insulin resistance, a positive association with HDL-C, and a U-shaped relationship with LDL-C. These associations varied by race/ethnicity and BMI, highlighting the presence of population-specific modifiers in the relationship between vitamin D status and metabolic outcomes.

The results underscore the potential clinical relevance of monitoring and addressing vitamin D deficiency, particularly among women with severely low 25(OH)D concentrations (<25.0 nmol/l), as part of post-GDM metabolic risk management. These findings contribute to a more nuanced understanding of the role of vitamin D in metabolic regulation and may inform the development of individualized intervention strategies for women with a history of GDM.

## Figures and Tables

**Table 1:** Baseline characteristics of pregnant women with gestational diabetes mellitus according to serum 25(OH)D concentrations in NHANES 2007–2018.

			Serum 25(OH)D concentrations (nmol/l)*			
	Total	<25.0	25.0–49.9	50.0–74.9	≥75.0	p-value
*Number of patients*	1876	312	643	576	345	
*Age ( mean ± SE), years*	35.93±0.11	35.78±0.28	35.86±0.18	35.81±0.21	36.15±0.25	0.756
*BMI*	31.76±0.18	34.78±0.44	32.67±0.28	31.84±0.34	29.52±0.37	<0.001
*Family income-poverty ratio*	2.80±0.04	2.17±0.08	2.38±0.06	2.85±0.07	3.37±0.09	<0.001
*Race*						<0.001
*Mexican American*	418 (12.70)	134 (21.83)	140 (22.46)	96 (9.72)	48 (4.19)	
*Non-Hispanic Black*	352 (10.70)	160 (26.62)	76 (10.27)	62 (7.91)	54 (4.47)	
*Non-Hispanic White*	642 (60.58)	76 (32.08)	130 (49.02)	186 (63.90)	250 (80.42)	
*Other Hispanic*	216 (6.09)	58 (9.24)	58 (7.59)	52 (5.93)	48 (3.61)	
*Other Race-Including Multi-Racial*	248 (9.93)	44 (10.23)	62 (10.67)	80 (12.54)	62 (7.30)	
*Drinking*						0.234
*No*	739 (35.73)	197 (39.57)	179 (34.61)	198 (38.62)	165 (32.09)	
*Yes*	1,137 (64.27)	275 (60.43)	287 (65.39)	278 (61.38)	297 (67.91)	
*Smoking*						0.294
*Always*	784 (40.85)	201 (38.15)	213 (46.36)	196 (43.12)	174 (37.26)	
*Ever*	949 (51.11)	239 (51.67)	213 (45.97)	233 (47.43)	264 (56.75)	
*Seldom*	143 (8.03)	32 (10.18)	40 (7.67)	47 (9.45)	24 (6.00)	
*Education levels*						0.009
*9*–*11^th^ Grade (Includes 12^th^ grade with no diploma)*	274 (11.90)	66 (14.28)	88 (16.32)	46 (6.86)	74 (11.84)	
*College Graduate or above*	376 (27.51)	66 (14.70)	76 (22.38)	118 (29.00)	116 (36.40)	
*High School Grad/GED or Equivalent*	358 (19.61)	96 (19.85)	86 (19.50)	92 (21.94)	84 (17.74)	
*Less Than 9* *^th^* * Grade*	196 (5.72)	58 (8.76)	62 (8.32)	48 (4.92)	28 (3.14)	
*Some College or AA degree*	672 (35.26)	186 (42.41)	154 (33.47)	172 (37.28)	160 (30.88)	
*Sport*						0.005
*No*	1,618 (82.42)	414 (88.99)	418 (87.44)	412 (83.72)	374 (74.82)	
*Yes*	258 (17.58)	58 (11.01)	48 (12.56)	64 (16.28)	88 (25.18)	
*Number of live births*						0.374
*<=3*	1,426 (80.90)	349 (78.51)	340 (77.42)	376 (86.20)	361 (80.18)	
*3*–*5*	337 (15.73)	86 (16.80)	100 (18.72)	74 (11.22)	77 (16.83)	
*>5*	113 (3.38)	37 (4.70)	26 (3.86)	26 (2.58)	24 (2.99)	

Data are numbers (percentages). All estimates accounted for complex survey designs.

* Vitamin D status was categorized into four groups: severe deficiency (<25.0 nmol/l), moderate deficiency (25.0–49.9 nmol/l), insufficient (50.0–74.9 nmol/l), and sufficient (≥75.0 nmol/l)according to the Endocrine Society Clinical Practice Guidelines.

**Table 2:** Least-squares means of glucose and lipid metabolism markers by serum 25(OH)D concentrations in women with gestational diabetes, NHANES 2007–2018.

	Serum 25(OH)D concentrations (nmol/l)				
	<25.0	25.0–49.9	50.0–74.9	≥75.0	p-value
*Glucose, mmol/l*	7.14±0.14	7.09±0.08	6.66±0.05	6.78±0.09	0.398
*HOMA-IR*	6.32±0.32	5.92±0.24	4.89±0.11	4.93±0.20	0.006
*Total cholesterol, mmol/l*	6.37±0.09	6.23±0.06	5.85±0.04	5.88±0.06	0.006
*HDL, mg/dl*	48.24±0.75	50.04±0.53	54.17±0.58	57.26±0.89	<0.001
*LDL, mmol/l*	2.93±0.03	3.01±0.02	3.02±0.02	3.01±0.03	0.488
*Triglyceride, mmol/l*	1.90±0.20	1.77±0.08	1.49±0.02	1.60±0.03	0.115

The least squares (mean ± SE) was estimated using general linear model with adjustment of age (continuous) and race/ethnicity (non-Hispanic white, or other), BMI, education level (less than high school, high school or equivalent, or college or above), drinking, smoking, Number of live births etc.

**Table 3:** Multivariable linear regression analysis of fasting glucose levels according to serum 25(OH)D concentrations.

	Serum 25(OH)D concentrations (nmol/l)				

	<25.0	25.0–49.9	50.0–74.9	≥75.0	p-value
*Glucose, mmol/l*					

Model 1* *β (95 % CI)^2^*	Reference	−0.05(−0.50, 0.40)	−0.49 (−0.89, −0.0)	−0.36 (−0.87, 0.14)	0.042
*Model 2*† *β (95 % CI)^2^*	Reference	0.04 (−0.44, 0.53)	−0.35(−0.80, 0.10)	−0.19 (−0.77, 0.40)	0.315
*Model 3*‡ *β (95 % CI)^2^*	Reference	0.02 (−0.42, 0.53)	−0.34(−0.80, 0.12)	−0.21 (−0.80, 0.39)	0.458

*HOMA-IR*					

Model 1* *β (95 % CI)^2^*	Reference	−0.40(−1.50, 0.70)	−1.43(−2.34,−0.5)	−1.39 (−2.35, −0.43)	<0.001
*Model 2*† *β (95 % CI)^2^*	Reference	−0.23(−1.43, 0.97)	−1.21(−2.30, 0.12)	−1.06 (−2.28, 0.16)	0.125
*Model 3*‡ *β (95 % CI)^2^*	Reference	−0.22(−1.40, 0.95)	−1.19(−2.28,−0.1)	−1.06 (−2.31, 0.18)	0.028

*Total cholesterol, mmol/l*					

Model 1* *β (95 % CI)^2^*	Reference	−0.13(−−0.45, 0.19)	−0.52(−0.79,−0.2)	−0.49 (−0.75, −0.22)	<0.001
*Model 2*† *β (95 % CI)^2^*	Reference	0.01 (−0.31, 0.32)	−0.29(−0.57,−0.0)	−0.26 (−0.56, 0.04)	0.038
*Model 3*‡ *β (95 % CI)^2^*	Reference	0.00(−0.32, 0.31)	−0.29(−0.57,−0.0)	−0.25 (−0.55, 0.05)	0.041

*HDL, mg/dl*					

Model 1* *β (95 % CI)^2^*	Reference	1.81 (−1.36, 4.97)	5.94 (2.86, 9.02)	9.02 (5.55, 12.50)	<0.001
*Model 2*† *β (95 % CI)^2^*	Reference	1.93 (−1.11, 4.97)	5.59 (2.47, 8.71)	7.96 (4.31, 11.61)	<0.001
*Model 3*‡ *β (95 % CI)^2^*	Reference	2.11 (−0.77, 4.99)	5.60 (2.58, 8.62)	7.47 (4.01, 10.93)	<0.001

*LDL, mmol/l*					

Model 1* *β (95 % CI)^2^*	Reference	0.08 (−0.07, 0.22)	0.09 (−0.07, 0.25)	0.08 (−0.05, 0.21)	0.654
*Model 2*† *β (95 % CI)^2^*	Reference	0.08 (−0.07, 0.23)	0.08 (−0.08, 0.24)	0.09 (−0.07, 0.24)	0.681
*Model 3*‡ *β (95 % CI)^2^*	Reference	0.08 (−0.07, 0.22)	0.08 (−0.08, 0.24)	0.10 (−0.05, 0.24)	0.612

*Triglyceride, mmol/l*					

Model 1* *β (95 % CI)^2^*	Reference	−0.12(−0.68, 0.44)	−0.41(−0.91, 0.09)	−0.29 (−0.79, 0.20)	0.341
*Model 2*† *β (95 % CI)^2^*	Reference	−0.17(−0.72, 0.38)	−0.46(−0.97, 0.05)	−0.40 (−0.90, 0.11)	0.245
*Model 3*‡ *β (95 % CI)^2^*	Reference	−0.18(−0.74, 0.39)	−0.46(−0.99, 0.07)	−0.39 (−0.89, 0.11)	0.211

2: β, beta coefficient; CI, Confidence Interval.

* Model 1: Crude model, unadjusted.

† Model 2: Adjusted for age, race/ethnicity, education level, and family income-to-poverty ratio.

‡ Model 3: Adjusted for all covariates in Model 2 plus physical activity, smoking status, alcohol consumption, and other clinical factors (HC>5).

**Table 4:** Stratified analysis of the association between serum 25(OH)D concentrations and metabolic markers in women with a history of gestational diabetes.

*Characteristic*	N	HOMA-IR	p-value	Glucose(mmol/l)	p-value	Total cholesterol(mmol/l)	p-value	HDL(mg/dl)	p-value	LDL(mmol/l)	p-value	Triglyceride(mmol/l)	p-value
*Overall*	1,876	*0.017 (−0.026, −0.007)		−0.006 (−0.010, −0.002)		−0.007 (−0.009, −0.004)		0.127 (0.102, 0.153)		0.000 (−0.001, 0.001)		−0.004 (−0.007, 0.000)	
*Race*			0.279		0.420		0.074		0.212		0.015		0.079
*Mexican American*	418	−0.020 (−0.046, 0.007)		0.004 (−0.007, 0.016)		−0.001 (−0.009, 0.007)		0.092 (0.026, 0.159)		0.003 (0.000, 0.006)		0.000 (−0.002, 0.003)	
*Non-Hispanic Black*	352	0.009 (−0.023, 0.042)		−0.005 (−0.005, 0.006)		−0.003 (−0.011, 0.005)		0.108 (0.048, 0.167)		−0.001 (−0.004, 0.002)		−0.003 (−0.005, 0.000)	
*Non-Hispanic White*	642	−0.020 (−0.035, −0.005)		−0.006 (−0.011, −0.001)		−0.004 (−0.007, 0.000)		0.159 (0.115, 0.202)		0.000 (−0.002, 0.002)		−0.007 (−0012, −0.001)	
*Other Hispanic*	216	−0.016 (−0.0.9, 0.008)		−0.007 (−0.024, 0.010)		−0.012 (−0.023, −0.001)		0.195 (0.112, 0.278)		0.001 (−0.005, 0.007)		−0.018 (−0.040, 0.005)	
*Other race including multi-racial*	248	−0.019 (−0.035, −0.004)		0.001 (−0.005, 0.007)		0.005 (0.000, 0.009)		0.140 (0.072, 0.208)		−0.005 (−0.008, −0.002)		−0.003 (−0007, 0.001)	
*Education*			0.027		0.088		0.787		0.045		0.347		0.004
*9–11* *^th^* * grade (Includes 12* *^th^* * grade with no diploma)*	274	−0.019 (−0.031, −0.006)		−0.005 (−0.012, 0.003)		−0.004 (−0.009, 0.001)		0.180 (0.123, 0.238)		0.000 (−0.002, 0.003)		−0.004 (−0.014, 0.007)	
*College graduate or above*	376	−0.033 (−0.065, −0.001)		−0.002 (−0.007, 0.004)		−0.007 (−0.013, −0.002)		0.140 (0.085, 0.196)		−0.002 (−0.004, 0.000)		−0.001 (−0.003, 0.002)	
*High School grade/GEDor equivalent*	358	−0.042 (−0.072, −0.013)		−0.015 (−0.026, −0.005)		−0.008 (−0.014, −0.001)		0.149 (0.086, 0.212)		−0.001 (−0.004, 0.001)		−0.003 (−0.008, 0.002)	
*Less than 9* *^th^* * grade*	196	−0.022 (−0.042, −0.003)		−0.010 (−0.034, 0.014)		−0.009 (−0.024, 0.006)		0.072 (−0.034, 0.178)		0.001 (−0.005, 0.007)		−0.028 (−0.060, 0.005)	
*Some college or AA degree*	672	0.000 (−0.012, 0.012)		0.000 (−0.006, 0.006)		−0.004 (−0.008, 0.000)		0.079 (0.039, 0.119)		0.001 (−0.001, 0.003)		0.000 (−0.002, 0.002)	
*BMI*			0.075		<0.001		0.013		0.605		0.114		0.128
*<25*	328	−0.008 (−0.016, −0.001)		−0.003 (−0.008, 0.003)		−0.005 (−0.009, 0.000)		0.114 (0.057, 0.171)		−0.002 (−0.003, 0.000)		0.000 (−0.002, 0.002)	
*25–30*	490	0.014 (0.005, 0.024)		0.002 (−0.004, 0.009)		0.000 (−0.005, 0.005)		0.083 (0.026, 0.140)		0.001 (−0.002, 0.003)		0.003 (0.001, 0.005)	
*>=30*	1046	−0.018 (−0.035, 0.000)		−0.005 (−0.001, 0.001)		−0.006 (−0.010, −0.002)		0.079 (0.049, 0.110)		0.001 (−0.001, 0.002)		−0.021 (−0.037, −0.005)	
*Drinking*			0.582		0.766		0.908		0.531		0.028		0.814
*No*		−0.020 (−0.035, −0.005)		−0.005 (−0.011, 0.001)		−0.007 (−0.011, −0.002)		0.138 (0.098, 0.177)		−0.002 (−0.003, 0.000)		−0.004 (−0.010, 0.001)	
*Yes*		-0.015 (−0.027, −0.002)		−0.006 (-0.011, −0.001)		−0.007 (−0.010, -0.003)		0.121 (0.088, 0.154)		0.001 (−0.001, 0.002)		−0.003 (−0.008, 0.001)	
*Sport*			0.256		0.528		0.803		0.954		0.421		0.624
*No*		−0.014 (−0.024, −0.003)		−0.006 (−0.010, −0.001)		−0.006 (−0.009, −0.003)		0.119 (0.092, 0.146)		0.000 (−0.001, 0.001)		-0.004 (− −0.008, 0.000)	
*Yes*		−0.028 (− −0.054, −0.003)		−0.003 (−0.009, 0.003)		−0.005 (−0.009, −0.001)		0.117 (0.050, 0.184)		−0.001 (−0.003, 0.001)		−0.002 (−0.004, 0.000)	
*Number of live births*			0.994		0.609		0.645		<0.001		0.455		0.433
*<=3*		−0.016 (−0.027, −0.005)		−0.006 (−0.010, −0.001)		−0.006 (−0.009, −0.003)		0.122 (0.093, 0.151)		−0.001 (−0.002, 0.001)		−0.004 (−0.008, 0.000)	
*3–5*		−0.017 (−0.042, 0.007)		−0.007 (−0.014, 0.001)		−0.008 (−0.014, −0.001)		0.190 (0.134, 0.246)		0.003 (−0.002, 0.007)		−0.006 (−0.014, 0.003)	
*>5*		−0.018 (−0.067, 0.032)		0.004 (−0.025, 0.033)		−0.001 (−0.016, 0.014)		−0.089 (−0.198, 0.020)		0.000 (−0.002, 0.003)		0.006 (0.000, 0.012)	
*Smoking*			0.025		0.151		0.988		0.011		0.124		0.168
*Always*		−0.001 (−0.019, 0.016)		−0.007 (−0.012, −0.001)		−0.007 (−0.011, −0.002)		0.085 (0.046, 0.124)		0.001 (−0.001, 0.003)		−0.006 (−0.013, 0.001)	
*Ever*		−0.024 (−0.036, −0.013)		−0.003 (−0.009, 0.002)		−0.007 (−0.011, −0.003)		0.164 (0.130, 0.198)		−0.001 (−0.002, 0.000)		−0.002 (−0.005, 0.001)	
*Seldom*		−0.015 (−0.089, −0.002)		−0.018 (−0.034, −0.002)		−0.007 (−0.019, 0.005)		0.099 (−0.007, 0.206)		−0.001 (−0.004, 0.003)		−0.004 (−0.012, 0.004)	

Data are presented as β (95 % CI), representing the change in the metabolic marker for each 1 nmol/l increase in serum 25(OH)D concentration. All models were adjusted for age, race/ethnicity, education level, body mass index (BMI), smoking status, alcohol consumption, and number of live births, unless the variable was used for stratification. The P for interaction was calculated by including a multiplicative interaction term between serum 25(OH)D (as a continuous variable) and the stratification variable in the corresponding multivariable linear regression model. Abbreviations: 25(OH)D, 25-hydroxyvitamin D; β, beta coefficient; CI, confidence interval; HOMA-IR, homeostasis model assessment of insulin resistance; HDL, high-density lipoprotein; LDL, low-density lipoprotein.
